# Hospitals, &c., of the City of New York

**Published:** 1859-09

**Authors:** 


*Hospitals, etc., of the City of New York.*—With this number we commence our series of articles on the Hospitals, etc., of New York. As is mentioned in the introduction to the first article, on the institutions for children, furnished by Prof. Austin Flint, it is designed to give, in this series, a correct idea of the immense clinical advantages at the command of students and practitioners in the Metropolis. These are so great, and so little appreciated abroad, that the accounts which we propose to give cannot fail to be of great use. The next in series will be an account of Ward's Island, which will be prepared by J. Carey Selden, M. D., one of the resident physicians. Following this will be an account of Blackwell's Island. Dr. Sanger, the distinguished resident physician, author of a late work on the history, etc., of prostitution, has kindly consented to be responsible for this article. Edward B. Dalton, M. D., of Bellevue Hospital, will furnish an article on that institution. Accounts of the remaining hospitals and of the dispensaries will follow ; which, though not yet provided for, we will obtain from men as well qualified as the above accomplished gentlemen to produce instructive and readable articles.

